# The impact of prothrombin complex concentrates when treating DOAC-associated bleeding: a review

**DOI:** 10.1186/s12245-018-0215-6

**Published:** 2018-12-03

**Authors:** Maureane Hoffman, Joshua N. Goldstein, Jerrold H. Levy

**Affiliations:** 1 Department of Pathology, Duke University School of Medicine, Durham, NC USA; 2 Department of Emergency Medicine, Massachusetts General Hospital, Boston, USA; 3 Department of Anesthesiology and Critical Care, Duke University School of Medicine, Durham, NC USA

**Keywords:** Anticoagulants, Haemorrhage, Non-vitamin K antagonist oral anticoagulants, Dabigatran, Rivaroxaban, Edoxaban, Apixaban, Prothrombin complex concentrates, Anticoagulant reversal

## Abstract

**Background:**

Bleeding complications are a risk associated with all anticoagulants. Currently, the treatment options for the management of direct oral anticoagulant (DOAC)-associated bleeding are limited. Prothrombin complex concentrates (PCCs) have been proposed as a potential therapeutic option, and evidence regarding their use is increasing.

**Review:**

Many studies supporting PCC have used preclinical models and healthy volunteers; however, more recently, observational studies have further improved insight into current DOAC reversal strategies. Multiple clinical practice guidelines now specifically suggest use of PCCs for this indication. Specific reversal agents for Factor Xa inhibitors may become available in the near future, but data on their efficacy are still emerging.

**Conclusions:**

Ultimately, a multimodal approach may be the optimal strategy to restore haemostasis in patients presenting with DOAC-associated coagulopathy.

**Electronic supplementary material:**

The online version of this article (10.1186/s12245-018-0215-6) contains supplementary material, which is available to authorized users.

## Background

**Fig. 1:**
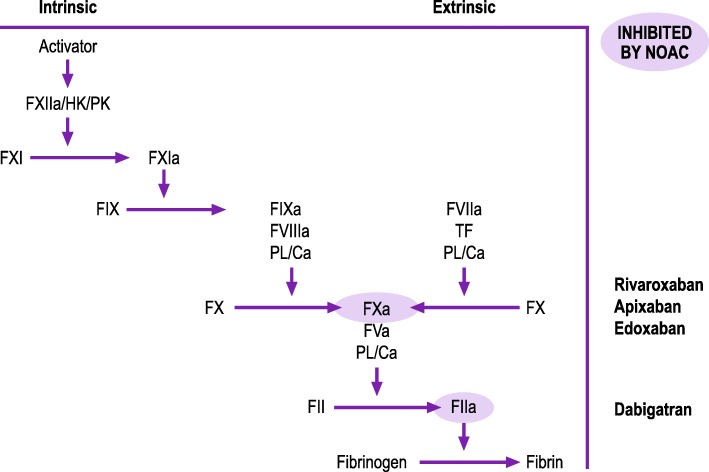
Effect of DOACs on the coagulation cascade. Figure legend: F, factor; Ca; calcium; HK, high-molecular-weight kininogen; DOAC, non-vitamin K antagonist oral anticoagulant; PL, phospholipid; PK, prekallikrein; TF, tissue factor

Direct oral anticoagulants (DOACs; also known as non-vitamin K oral anticoagulants or target-specific oral anticoagulants) specifically inhibit either coagulation factor IIa (FIIa; dabigatran) or factor Xa (FXa; rivaroxaban, apixaban and edoxaban; Fig. 1) [1]. DOAC use has grown in recent years, especially for stroke prevention in patients with non-valvular atrial fibrillation [1].

Most evidence suggests rates of major bleeding and intracranial haemorrhage (ICH) with DOACs are similar to or lower than those with warfarin [2], although some studies have highlighted a higher rate of gastrointestinal bleeding with particular DOACs [2, 3]; high-dose dabigatran and high-dose edoxaban were associated with an increased risk of gastrointestinal bleeding versus warfarin (*p* < 0.001 and *p* = 0.03 respectively) [4, 5], and rivaroxaban was associated with an increased incidence of major bleeding from a gastrointestinal site versus warfarin (3.2% vs 2.2%; *p* < 0.001) [6]. Rates of major bleeding for dabigatran, rivaroxaban, apixaban and edoxaban (as compared with warfarin) are listed in Table 1 [7].

**Table 1:** Comparative bleeding rates for warfarin versus dabigatran, rivaroxaban, apixaban and edoxaban [7]

	RE-LY [5]			ROCKET-AF [6]		ARISTOTLE [136]		ENGAGE-AF TIMI [4]		
	Dabigatran 150 mg (*n* = 6076)	Dabigatran 110 mg (*n* = 6015)	Warfarin (*n* = 6022)	Rivaroxaban (*n* = 7111)	Warfarin (*n* = 7125)	Apixaban (*n* = 9088)	Warfarin (*n* = 9052)	Edoxaban 60 mg (*n* = 7012)	Edoxaban 30 mg (*n* = 7002)	Warfarin (*n* = 7012)
Major bleeding rate (%) per year	3.11	2.71	3.36	3.6	3.4	2.13	3.09	2.75	1.61	3.43
Hazard ratio vs warfarin	0.93 (0.81–1.07); *p* = 0.31	0.80 (0.69–0.93); *p* = 0.003	/	1.04 (0.90–1.20); *p* = 0.58	/	0.69 (0.60–0.80); *p* < 0.001	/	0.80 (0.71–0.91); *p* < 0.001	0.47 (0.41–0.55); *p* < 0.001	/

**Fig. 2:**
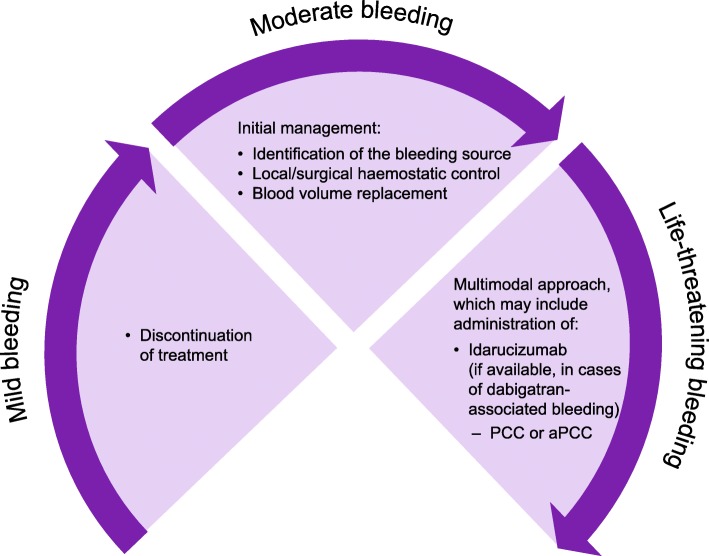
Treatment of DOAC-associated bleeding. Figure legend: aPCC, activated prothrombin complex concentrate; DOAC, non-vitamin K antagonist oral anticoagulant; PCC, prothrombin complex concentrate

Guidance for the management of DOAC-associated bleeding is summarised in Fig. 2 and includes discontinuation of DOAC treatment (may be sufficient in cases of mild bleeding), local/surgical haemostatic control and blood volume replacement. However, these measures may not be sufficient to control bleeding and restore haemostasis in life-threatening situations.

Prothrombin complex concentrates (PCCs) routinely contain high concentrations of factors II, IX and X (three-factor [3F] PCCs) and potentially also high concentrations of factor VII (four-factor [4F] PCCs) [8]. These high clotting factor concentrations mean PCC administration may overcome the anticoagulant effects of DOAC administration by enhancing thrombin generation [9]. PCCs generally contain nonactivated clotting factors, but PCCs including activated factor VII (in conjunction with non-activated factors II, IX and X) are also available; these PCC types are referred to as nonactivated and activated PCCs respectively [10].

4F-PCCs are the established first-choice agent for urgent vitamin K antagonist (VKA) reversal in cases of major bleeding or prior to emergency surgery [11]. However, management of DOAC-associated bleeding remains a clinical challenge, especially with FXa inhibitors, for which there is only one specific reversal agent (Andexanet alfa) available [12].

PCCs have been extensively studied for their potential to correct DOAC-associated coagulopathy, but until recently, clinical data were limited. Key preclinical studies carried out prior to 2013 are described in our previous review [8]. Here, we aim to review more recent evidence and discuss the potential therapeutic role of PCCs in the treatment of DOAC-associated bleeding. Data for review were identified using the literature search strategy described in Additional file 1.

## Clinical relevance of laboratory assays

Many studies evaluating the effects of haemostatic agents on DOAC-mediated anticoagulation have focused on effects on laboratory parameters.

### Traditional coagulation assays

Traditional coagulation assays include prothrombin time (PT), activated partial prothromboplastin time (aPTT), international normalised ratio (INR), and thrombin time (TT). PT and aPTT are among the most commonly used screening tests for coagulation abnormalities and reflect the activity of the extrinsic and intrinsic coagulation pathways, respectively; INR is the standardised ratio of the patient’s PT and a normal PT value [13]. TT measures the fibrin polymerisation process following the addition of thrombin and is sensitive to the presence of thrombin inhibitors, such as heparin [14]. Although these assays provide information on clotting factor levels, overall they are poor predictors of bleeding [13].

The INR is used to monitor the degree of anticoagulation in VKA-treated patients and is not suitable for measuring the effect of DOACs [15]. The direct FXa inhibitors may prolong the PT, but the extent of prolongation is variable; impact varies by FXa inhibitor, thromboplastin reagent used and also the health status of the patient [16]. Other assays are generally not sensitive to these agents [15]. The direct thrombin inhibitor, dabigatran, prolongs aPTT (depending on the reagent/instrument system used) [17] and correlates with other assays when at therapeutic levels [18]. Specific assays, including calibrated anti-FXa assays for FXa inhibitors [15], and diluted TT (dTT) and ecarin clotting time (ECT; an assay that measures time to clot formation using ecarin as a reagent) for dabigatran [18], may be appropriate for monitoring the effects of DOACs, especially at lower plasma levels.

### Thrombin generation assays

While traditional assays reflect the formation of a fibrin clot, the thrombin generation assay (TGA) corresponds to the overall function of the coagulation system [13]. In this assay, the time course of thrombin generation is represented graphically, from which various parameters can be derived: lag time (the time until a measurable amount of thrombin is formed), peak height (the maximum level of thrombin attained), time to peak and endogenous thrombin potential (ETP; total amount of thrombin formed, also described as the area under the curve). It has been suggested that TGAs show greater correlation with the degree of haemostatic impairment resulting from DOACs in vivo than other assays [8, 19, 20]. However, not all TGA results are comparable. The performance characteristics of TGAs depend on the agent used to initiate thrombin generation [19, 21] (for example, tissue factor or phospholipids), and results have historically been difficult to standardise between laboratories.

### Viscoelastic tests

Thromboelastography (TEG) and rotational thromboelastometry (ROTEM) assays provide information on clot formation and dissolution, allowing measurement of the speed of clot formation, clot strength and clot lysis, among other characteristics [13]; both techniques have been shown to be of practical and diagnostic use in acute traumatic coagulopathy settings, but require further validation regarding therapeutic use [22]. Viscoelastic testing has not yet been proven to be useful for DOAC detection or reversal in general clinical practice, although preliminary testing of a commercial rapid TEG assay in healthy volunteers showed that detection and monitoring of rivaroxaban, apixaban and dabigatran is possible with this method [23]. New reagents are also being developed to facilitate these analyses [24, 25].

### Clinical relevance of laboratory assays: Summary

Effects on laboratory parameters do not necessarily correlate with bleeding measures [19, 20, 26–29], although some studies suggest that ETP may be an appropriate surrogate biomarker for assessing effects on bleeding [19, 30]. In addition, some of these tests are not available for use in emergency situations. Ultimately, studies evaluating the effect of various reversal agents on bleeding, rather than laboratory parameters, have more clinical relevance for evaluating treatment of DOAC-associated bleeding.

## Overview of recent data (2013–2017)

A review and discussion of the most recent data are presented below. A limitation of many of the studies is that they were conducted in animal models or healthy volunteers.

### Preclinical data

#### Animal studies

A number of studies have examined DOAC reversal in animals (Additional file 2) [19, 20, 28, 29, 31–38]. Most found improvements in bleeding parameters after PCC treatment. Moreover, studies conducted using a pig polytrauma model also demonstrated an improved survival rate in dabigatran-treated animals administered 4F-PCC or activated PCC (aPCC) compared with controlled-treated animals [34, 35, 38]. Effects on laboratory parameters, however, were inconsistent; of the assays employed, TGAs had the best correlation with bleeding parameters.

#### In vitro and ex vivo human studies

The potential role for PCCs in treating DOAC-associated bleeding has also been evaluated in studies using blood samples from patients and healthy volunteers (Additional file 3) [21, 39–56]. PCCs reversed some of the effects of DOACs on laboratory assays, including in two studies which utilised TEG and/or ROTEM to assess the impact of dabigatran/apixaban [41, 54]. However, these effects on laboratory assays do not necessarily correlate with bleeding.

### Healthy volunteers

Several randomised trials have evaluated the effect of PCCs on DOAC-treated healthy volunteers (Table 2), but most assessed effects on laboratory parameters rather than bleeding.

**Table 2:** Randomised studies in healthy volunteers

Study	Design	N	DOAC	PCC	Results		
					Laboratory parameters tested	Reversal of DOAC effects with PCC	Bleeding
Barco et al. [58]	Randomised, double-blind, placebo-controlled, crossover	6	Rivaroxaban (15 mg BID)	4F-PCC (Cofact® 25 or 37.5 IU/kg)	PT	(+) with both doses of 4F-PCC	No deaths, SAEs or TEEs reported
					ETP	(+) with 4F-PCC 37.5 IU/kg	
Brown et al. [60]	Randomised, double-blind, placebo-controlled, three-way crossover	24	Edoxaban (60 or 120 mg)	3F-PCC (Bebulin® 25 or 50 IU/kg)	PT	(−)	No deaths, SAEs or TEEs reported
					ETP	(+)	
Cheung et al. [61]	Randomised, double-blind, placebo-controlled, crossover	6	Apixaban (10 mg BID)	4F-PCC (Cofact® 25 or 37.5 IU/kg)	PT	(+)	No deaths, SAEs or TEEs reported
					ETP	Partial	
Eerenberg et al. [9]	Randomised, double-blind, placebo-controlled, crossover	12	Dabigatran (150 mg BID)	4F-PCC (Cofact® 50 IU/kg)	TGA lag time, aPTT, ECT, TT	(−)	No deaths, SAEs or TEEs reported
			Rivaroxaban (20 mg BID)		PT, ETP	(+)	
Levi et al. [57]	Randomised, open-label, parallel-group	35	Rivaroxaban (20 mg BID)	4F-PCC (Beriplex® 50 IU/kg) and 3F-PCC (Profilnine® 50 IU/kg)	PT	(+) 4F-PCC > 3F-PCC	No deaths, SAEs or TEEs reported
					TGA		
					ETP	(+) 3F-PCC > 4F-PCC	
					Peak TG	(+) with 3F-PCC only	
					Time to peak	(+)	
					Lag time	(-)	
					aPTT	(-)	
Levy et al. [59]	Randomised, double-blind, parallel-group	147	Rivaroxaban (20 mg BID)	4F-PCC (Kcentra® 50 IU/kg)	PT	Partial	No deaths, SAEs or TEEs reported; no difference from saline control in reversal of effects on bleeding duration and volume after administration of 4F-PCC or TXA
					TGA		
					ETP	(+)	
Nagalla et al. [63]	Randomised, two-period crossover, assessor-blinded	12	Apixaban (5 mg BID)	4F-PCC (Kcentra®/Beriplex® 25 IU/kg)	PT	(+)	No deaths, SAEs or TEEs reported
					TGA parameters (peak, lag time, ETP)	(+) (significan\ce dependent on reagents used)	
					TGA parameters (time to peak, maximum velocity)	(-)	
					aPTT	(-)	
Song et al. [62]	Open-label, randomised, placebo-controlled, 3-period crossover	15	Apixaban (10 mg BID)	4F-PCC (Beriplex® or Cofact® 50 IU/kg)	TGA	(+)	No deaths, SAEs or TEEs reported
					ETP	(+)	
					Peak height	(-)	
					Lag time	(-)	
					Time to peak	(-)	
					Velocity index	(+)	
					PT		
Zahir et al. [30]	Randomised, double-blind, placebo-controlled, two-sequence, two-period, crossover	93	Edoxaban (60 mg)	4F-PCC (Beriplex® 10, 25 or 50 IU/kg)	PT	Partial with 4F-PCC 50 IU/kg	No deaths, SAEs or TEEs reported; reversal of effects on bleeding duration and volume (with 50 IU/kg; partial reversal with 25 IU/kg)
					ETP	(+) with 50?IU/kg (partial with 25?IU/kg)	

(−), negative result; (+), positive result. *3F-PCC* three-factor prothrombin complex concentrate, *4F-PCC* four-factor prothrombin complex concentrate, *aPCC* activated prothrombin complex concentrate, *aPTT* activated partial thromboplastin time, *BID bis in die* (twice daily), *ECT* ecarin clotting time, *ETP* endogenous thrombin potential, *PT* prothrombin time, *TG* thrombin generation, *TGA* thrombin generation assay, *TT* thrombin time, *TXA* tranexamic acid

#### Laboratory parameters

##### Dabigatran and rivaroxaban

In a randomised, double-blind, placebo-controlled crossover study, administration of 4F-PCC was insufficient to correct the effects of dabigatran on various laboratory parameters, but did reverse the effects of rivaroxaban on ETP [9].

##### Rivaroxaban

Three additional studies investigated the effect of PCCs on rivaroxaban-induced anticoagulation. In the first, a randomised, open-label, parallel-group study, 3F-PCC reversed the effects of rivaroxaban on TGA parameters (ETP and time to peak) to a greater extent than 4F-PCC [57]. The second, a randomised, double-blind, placebo-controlled, crossover trial, demonstrated a significant effect of a 4F-PCC (37.5 IU/kg) on rivaroxaban-induced ETP inhibition (*p* = 0.03) [58]. The third, a randomised, double-blind, parallel-group study, showed that 4F-PCC (50 IU/kg) partially reversed the effects of rivaroxaban on PT and completely reversed the effects on ETP within 30 min following end of infusion, whereas tranexamic acid (1.0 g) showed no difference from saline on either PT or ETP [59].

##### Edoxaban

A phase 1 study in healthy volunteers (*n* = 24) showed 3F-PCC rapidly and completely reversed the effect of edoxaban on ETP versus placebo but had no effect on edoxaban-mediated PT prolongation [60].

##### Apixaban

Three studies evaluated the effect of PCCs in apixaban-treated healthy volunteers. In a single-centre, randomised, double-blind, placebo-controlled, crossover study, apixaban produced significant modifications in ETP and PT (*p* < 0.01 for both) [61]. Subsequent 4F-PCC administration increased ETP and normalised PT. Similarly, in an open-label, randomised, placebo-controlled 3-period crossover study, 4F-PCC rapidly reversed apixaban-mediated effects on PT, and ETP and peak height, but not other TGA parameters [62]. In another randomised, two-period crossover, assessor-blinded trial, 4F-PCC improved the effects of apixaban on some TGA parameters [63].

#### Bleeding

To date, two studies have investigated the effect of PCCs on bleeding parameters. The first was a phase 1 study conducted in two parts [30]. Part 1 determined the appropriate dose of edoxaban and assessed intra-individual variability of bleeding duration and volume following a skin punch biopsy in edoxaban-treated healthy volunteers. Part 2 was a double-blind, randomised, placebo-controlled, two-sequence, two-period crossover study with a relatively large sample size (*n* = 93). Based on its lower intra-individual variability in part 1 of the study, bleeding duration rather than volume was the primary endpoint. 4F-PCC administration dose-dependently reversed the effects of edoxaban on bleeding duration. Complete reversal was achieved following administration of 4F-PCC 50 IU/kg, with partial reversal at a dose of 25 IU/kg and no reversal at 10 IU/kg. A similar trend was observed for bleeding volume and ETP. A dose-dependent reversal of PT was also observed, but this parameter was less well correlated with bleeding effects than ETP.

In the second study, bleeding duration and volume were assessed following administration of rivaroxaban 20 mg twice daily for 4 days and then following administration of 4F-PCC 50 IU/kg or tranexamic acid 1.0 g. For both 4F-PCC and tranexamic acid, no difference from a saline control was observed in either bleeding duration or volume, despite 4F-PCC demonstrating effects on PT and ETP [59].

#### Clinical trials in healthy volunteers: summary

Overall, as observed in the preclinical studies, PCC administration led to alterations in some, but not all, laboratory parameters affected by DOACs. Studies with the FXa inhibitors suggest that anticoagulation reversal can be demonstrated using ETP assays [9, 30, 59, 60]. A good correlation between effects on ETP and bleeding parameters was observed in the phase 1 study by Zahir and colleagues; this has also been demonstrated in preclinical studies in animal models [19, 31], suggesting that ETP could be an appropriate surrogate biomarker for assessing the effect of PCCs on bleeding, as mentioned previously.

Generally, PCCs were well-tolerated in healthy volunteers treated with DOACs; there were no reported thromboembolic events (TEEs), serious adverse events or deaths [9, 30, 57–61, 63]. However, it is important to consider that the risk of such events differs between healthy volunteers, patients with medical conditions requiring anticoagulation and patients suffering bleeding while receiving anticoagulants.

### Clinical data from DOAC-treated patients

Few studies of anticoagulation reversal strategies have been conducted in patients presenting with major bleeding or requiring urgent surgery. However, data on patient outcomes are starting to emerge from registries and other observational studies, as well as in case reports from clinical practice.

#### Registries and observational studies

##### Major bleeding

According to ISTH criteria, major bleeding in non-surgical patients is defined as:

1. Fatal bleeding
2. Symptomatic bleeding in a critical area or organ
3. Bleeding causing a fall in haemoglobin level of ≥ 20 g/L (≥ 1.24 mmol/L), or leading to transfusion of ≥ 2 units of whole blood or red cells [64]

Data from the large, prospective Dresden DOAC registry was used to analyse rates, management and outcomes of rivaroxaban-related bleeding in 1776 patients [65]. Sixty-six major bleeding events (defined according to ISTH criteria) were reported in 59 patients. Most cases were treated conservatively, with haemostatic intervention only considered necessary in nine patients, highlighting that reversal of DOAC-mediated anticoagulation is rarely needed. PCC was administered in six patients; time between admission and PCC administration was 1–22 h, and the dose was 18–47 IU/kg. Of the four cases for which coagulation tests were repeated after PCC administration, one patient showed improvement (INR, PT and aPTT). Haemorrhage stabilisation was reported in 5/6 patients; the remaining patient who did not respond received the lowest dose of PCC (18 IU/kg), suggesting that the effective dose may be greater than this. Although the most appropriate dose of PCC for treatment of DOAC-associated bleeding has not been established, preclinical studies suggest it to be 25–50 IU/kg.

The French Working Group on Perioperative Haemostasis (GIHP) has also conducted a large, prospective, multicentre registry to evaluate the management of major bleeding in 732 patients treated with dabigatran, rivaroxaban or apixaban [66]. Overall, 208/732 (28%) and 73/732 (10%) patients received 4F-PCC (either Confidex®/Beriplex®, Octaplex® or Kanokad®) and aPCC, respectively. Although the study was not designed to evaluate the efficacy of PCC, investigator-assessed haemostasis was totally or partially achieved in 44% and 37% of those who received these agents, respectively.

In another multicentre, prospective study (the Unactivated Prothrombin complex concentrates for the Reversal of Anti-factor Ten inhibitors [UPRATE] study) evaluating the use of 4F-PCC (Beriplex® or Octaplex®) for the management of rivaroxaban- or apixaban-associated major bleeding, haemostasis was judged effective in 58/84 (69%) patients [67].

A prospective, non-interventional, multicentre cohort study conducted in nine Canadian hospitals has evaluated the use of 4F-PCC (Beriplex® or Octaplex®) at a fixed dose of 2000 U for the management of major bleeding in 66 patients treated with rivaroxaban or apixaban [68]. Haemostatic effectiveness was assessed as good by the treating physician in 43 (65%) patients, moderate in 13 (20%) and poor/none in 10 (15%). It should be noted that there was a dose deviation in 12 (18%) patients.

Use of aPCC has been evaluated in a prospective study of 14 patients with dabigatran-associated bleeding [69]. The effectiveness of aPCC was considered by the treating physician to be good in nine (64%) patients and moderate in five (36%).

A further prospective, non-interventional study (the Reversal Agent use in patients treated with Direct Oral Anticoagulants or vitamin K antagonists [RADOA] registry) is currently being conducted in Germany to evaluate the reversal strategies used in patients treated with oral anticoagulants [70].

A small single-centre prospective trial monitoring laboratory parameters in 13 patients who received 4F-PCC (Beriplex®) following major bleeding (majority intra-cranial or subdural haemorrhage) found that the ETP and peak thrombin generation improved from baseline by 68% (*p* = 0.001) and 54% (*p* = 0.001), respectively, 10 min after completion of PCC treatment [71]. Improvements in several other laboratory parameters were observed and bleeding ceased in 77% of patients receiving 4F-PCC. No thromboses associated with treatment were identified at 7 days post administration [71].

Retrospective studies have also provided insight into DOAC-associated bleeding management in real-world settings. In a large chart review, PCCs (median dose 1500 IU) and aPCCs (median dose 3534 IU) were used in 57 (12.4%) and 24 (5.2%) out of 460 cases of DOAC-associated bleeding, respectively; however, clinical outcomes by treatment type were not reported [72]. In a retrospective review of 25 patients presenting with dabigatran- or rivaroxaban-associated haemorrhage, PCC (median dose 40 IU/kg) was administered in three cases (all rivaroxaban) [73]. Haemorrhages resolved in these cases. Based on data from this study, the institutions involved developed protocols for DOAC-associated haemorrhage, including use of PCCs for severe bleeding events. In another chart review, 28 patients received 4F-PCC (Kcentra®/Beriplex®) according to an institutional protocol designed for the treatment of FXa inhibitor-associated major bleeding or for those requiring emergency surgery [74]. One patient experienced a TEE; although the relationship between this event and 4F-PCC treatment was not established, the patient had a history of TEEs and was not re-anticoagulated after treatment. No data on bleeding outcomes were reported.

##### ICH

In cases of coagulopathic ICH, the hematoma may continue to expand for up to 24 h; it is therefore essential to apply coagulopathy reversal treatment promptly and at an effective dose in order to obtain the best outcome for the patient [75].

An analysis of data from the multicentre, prospective, observational Registry of Acute Stroke Under New Oral Anticoagulants (RASUNOA) was performed to evaluate the management of a subgroup of patients with DOAC-associated intracerebral haemorrhage (*n* = 61) [76]. Consistent with prescribing data [77], rivaroxaban was the most commonly used DOAC (80%), with 12% and 8% of patients receiving dabigatran and apixaban, respectively. 4F-PCC was administered to 35/61 (57%) patients (mean [SD] dose, 2390 [980] IU). When comparing patients who did/did not receive 4F-PCC, there was no significant difference with regard to early haematoma expansion (4F-PCC, 43%; no 4F-PCC, 29%; *p* = 0.53) or 3-month functional outcome. This may be attributed to delayed administration of 4F-PCC (which was often not administered for ≥ 5 h), or differences in patient baseline characteristics in each group; patients who received 4F-PCC generally had a worse clinical status, and more frequently had a deep haemorrhage location, than those who did not receive 4F-PCC. Timely intervention is thought to be critical in ICH cases [78] to limit haematoma expansion and improve patient outcomes.

The second part of the Germany-wide RETRACE study, a retrospective cohort analysis of 190 patients with DOAC-associated ICH, investigated the effect of PCC administration on haematoma enlargement [79]. Overall, PCC at any dose prior to follow-up imaging was not statistically significantly associated with a reduced risk of haematoma enlargement in the DOAC-ICH cohort (risk ratio (RR) = 1.150; 95% confidence interval (CI) 0.632–2.090). Importantly, the chance that the nonsignificant nature of these findings is a result of a power issue and small patient numbers cannot be excluded [79].

A single-centre retrospective analysis evaluated outcomes among 55 DOAC-anticoagulated patients presenting with ICH [80]. 4F-PCC (Beriplex® or Octaplex®) was administered in 31/55 (56%) cases (median dose 2000 IU). The 30-day mortality rate was 20%, which is slightly lower than the rate observed at discharge in the literature for patients with anticoagulation-related ICH [81].

In another retrospective study, 90-day outcomes were favourable in 6/18 (33%) patients treated with 4F-PCC (Kcentra®/Beriplex®) for FXa inhibitor-associated ICH, with the authors concluding that 4F-PCC appeared effective in reducing haematoma expansion in this small patient population [82].

Two small retrospective analyses investigated the effect of aPCC in patients presenting with DOAC-associated ICH [83, 84]. In one of the studies, 6/11 (55%) patients demonstrated stable ICH after aPCC administration [83], and in the other study, no cases of haematoma expansion occurred in the six patients who were previously receiving DOACs [84].

##### Periprocedural bleeding

In a retrospective, multicentre study, 4F-PCC (Kcentra®/Beriplex®) was part of the institutional protocol to mitigate the effects of FXa inhibitors [85]. Here, 4F-PCC was effective for the management of acute pericardial effusion in patients undergoing atrial fibrillation ablation while anticoagulated with rivaroxaban or apixaban.

#### Case studies/series

A number of case reports have discussed individualised treatment of DOAC-associated bleeding (Table 3) [27, 86–107]. The number and diversity of these reports suggest that many hospitals are adopting PCCs as part of treatment strategies for DOAC-associated bleeding.

**Table 3:** Case studies on the use of PCC for DOAC reversal, January 2013–February 2017

Study citation	Study setting	Cases (*N*)	DOAC	PCC used	Outcomes following PCC administration
Patients presenting with major bleeding					
Denetclaw et al. [96]	Gluteal arterial extravasation	1	Apixaban	3F-PCC (Profilnine®)	• Clinical examination suggested the haematoma had not expanded since PCC administration • The patient was discharged in a stable condition on Day 7 • No TEEs or VTEs reported
Diaz et al. [97]	GI bleeding	5	Dabigatran	4F-PCC (Octaplex®)	• Cessation of bleeding in 4/5 patients • No TEEs or VTEs reported during 6 months of follow-up
Dibu et al. [86]	ICH	5	Rivaroxaban, apixaban, dabigatran	aPCC (FEIBA®)	• None of the patients had ICH expansion • No TEEs, VTEs or haemorrhagic complications reported
Durie et al. [101]	Life-threatening bleed due to trauma	1	Apixaban	4F-PCC (Kcentra®/Beriplex®)	• Despite aggressive treatment, and PCC administered at the maximum dose, haemostasis was not achieved and the patient died
Faust et al. [102]	Subdural haematoma	3	Rivaroxaban	3F-PCC (Profilnine®)	• Haematoma volume remained stable in all patients • No TEEs or VTEs reported
Faust et al. [107]	Subdural haematoma	2	Apixaban	3F-PCC (Profilnine®)	• Minimal or no progression in haematoma volume • No TEEs or VTEs reported
Faust and Peterson [87]	Intracerebral haemorrhage	1	Dabigatran	aPCC (FEIBA®)	• Coagulation parameters were not normalised • Despite initial increase in haematoma, the patient avoided surgical intervention and remained stable • After approximately 2 weeks, the patient developed a new ischemic stroke and was discharged to a hospice
Jones et al. [104]	GI bleeding and haemorrhagic shock	1	Dabigatran	4F-PCC (Kcentra®/Beriplex®)	• Rapid correction of coagulation parameters and achievement of haemostasis • No TEEs or VTEs reported
Kauffmann et al. [98]	Subdural haematoma	1	Rivaroxaban	4F-PCC (Kanokad®)	• Improvement in TGA parameters lasting at least 18 h (normalisation of lag time, elevated peak height and ETP) • Favourable clinical outcome • No TEEs or VTEs reported
Masotti et al. [27]	GI bleeding	8	Dabigatran	4F-PCC (Confidex®/Beriplex®)	• Cessation of bleeding, despite uncorrected coagulation parameters (aPTT, PT/INR) • No TEEs or VTEs reported
McGovern et al. [99]	GI bleeding	1	Dabigatran	4F-PCC (unspecified)	• Normalisation of coagulation profile (PT, INR, aPTT) • No active sites of bleeding identified, and no further blood transfusions required • Patient discharged 4 days later, without experiencing any further morbidity related to his condition • No TEEs or VTEs reported
Means et al. [103]	Rectal bleeding	1	Rivaroxaban	3F-PCC (Profilnine®)	• PCC administration helped control bleeding • No TEEs or VTEs reported
Rinehart et al. [88]	Subdural haematoma	1	Apixaban	aPCC (FEIBA®)	• PT and INR normalised within 24 h • Slight improvement in subdural haematoma on Day 2 • Patient remained stable, with further improvement in the subdural haematoma before discharge on Day 7 • No TEEs or VTEs reported
Schulman et al. [100]	Subdural haematoma	1	Dabigatran	aPCC (FEIBA®)	• Normalisation of thrombin time at Day 3 • Patient underwent an uneventful haematoma drainage procedure and was discharged a day later • No TEEs or VTEs reported
	Intra-axial haemorrhage	1			• Mild increase in haematoma size after 3 days; no further progression of symptoms • No TEEs or VTEs reported
	Pericardial bleeding	1			• Cessation of bleeding • No TEEs or VTEs reported
	Upper GI bleeding	1			• Stabilisation of clinical condition • No TEEs or VTEs reported
Smith et al. [106]	Left frontal lobe parenchymal haemorrhage	1	Rivaroxaban	aPCC (FEIBA®)	• After aPCC administration, the patient was admitted to the trauma in-patient unit; neurological exam remained normal for 24 h and the patient was discharged • No TEEs or VTEs reported
Patients requiring urgent/emergent surgery					
Beynon et al. [89]	Emergency neurosurgery	2	Apixaban	4F-PCC (Beriplex®)	• No bleeding complications occurred during surgery • No symptoms suggestive of thromboembolic events
Chic Acevedo et al. [90]	Urgent surgery due to an abdominal haematoma	1	Rivaroxaban	4F-PCC (Octaplex®)	• PT normalisation • No complications reported during or after surgery
Dager et al. [95]	Ablation procedure	1	Dabigatran	aPCC (FEIBA®)	• Normalisation of INR and aPTT, but not thrombin time • Bleeding slowed 5 min into the infusion and had stopped by the end of the 15-min infusion • No evidence of thrombosis observed
Liu et al. [105]	Coronary artery bypass graft	1	Rivaroxaban	4F-PCC (Kcentra®/Beriplex®)	• Patient lost 650 mL blood during the procedure, necessitating the transfusion of 2 units each of PRBCs and platelets • Patient was discharged with no sequalae on postoperative day 9 • Follow-up visit at 1 month did not reveal any new significant events • No TEEs or VTEs reported
Maurice-Szamburski et al. [91]	Subdural haematoma requiring neurosurgical intervention	1	Rivaroxaban	aPCC (FEIBA®)	• No abnormal bleeding during surgery • Good surgical result with no rebleeding • No TEEs or VTEs reported
Neyens et al. [92]	Subdural haematoma requiring neurosurgical intervention	1	Dabigatran	aPCC (FEIBA®)	• Therapeutic impact uncertain • Coagulation parameters (aPTT and TT) remained prolonged, delaying surgical intervention; thromboelastography may be more appropriate for monitoring dabigatran anticoagulation • No TEEs or VTEs reported
Puttick et al. [93]	Emergency surgery for an incarcerated femoral hernia	1	Dabigatran	aPCC (FEIBA®)	• Surgery was successful, with no complications • The patient made an uneventful recovery and was discharged the next day • No TEEs or VTEs reported
Wong and Keeling [94]	Urgent percutaneous transhepatic drainage of a gall bladder empyema	1	Dabigatran	aPCC (FEIBA®)	• No bleeding complications occurred during surgery and the clinical state of the patient improved • No TEEs or VTEs reported

*3F-PCC* three-factor prothrombin complex concentrate, *4F-PCC* four-factor prothrombin complex concentrate, *aPCC* activated prothrombin complex concentrate, *aPTT* activated partial thromboplastin time, *ETP* endogenous thrombin potential, *INR* international normalised ratio, *PRBC* packed red blood cells, *PT* prothrombin time, *TGA* thrombin generation assay, *TEE* thromboembolic event, *TT* thrombin time, *VTE* venous thromboembolism

### Clinical practice guidelines

Based on the data currently available, many clinical practice guidelines suggest that PCC administration should be considered in cases of serious or life-threatening bleeding (Table 4) [78, 108–118]. This is also reflected in other expert proposals and recommendations [119–122], in DOAC prescribing information [123, 124] and in a recent clinical practice survey in the Netherlands, in which most of the 61 neurologist respondents used PCC for the treatment of intracerebral haemorrhage associated with factor IIa inhibitors (74%) or factor Xa inhibitors (72%) [125].

**Table 4:** Use of PCCs to reverse the anticoagulant effect of DOACs: summary of guidelines and proposals

Society/group (citation)	Need for DOAC reversal	Guidelines or proposals regarding the use of PCCs
Thrombosis and Hemostasis Summit of North America [113]	Critical bleeding or emergent surgery	• Use of PCCs seems to be a reasonable approach in dire clinical situations • Consensus was not reached because two authors believed that PCC could not be recommended due to absence of data at the time
Task Force for Advanced Bleeding Care in Trauma [117]	Life-threatening bleeding	• PCC (25–50 IU/kg) suggested for reversal of FXa inhibitors • Not suggested for patients treated with dabigatran
European Society of Cardiology; European Heart Rhythm Association [111, 112]	Life-threatening bleeding	• Administration of PCC (25 IU/kg, repeated if clinically indicated) or aPCC (50 IU/kg) can be considered
European Society of Anaesthesiology [114]	Life-threatening bleeding or ICH	• PCC or aPCC suggested
Australasian Society of Thrombosis and Haemostasis [118]	Life- or limb-threatening bleeding	• Reasonable to consider the use of PCCs; risk and benefit of these agents should be assessed in each individual patient
*Groupe d’Intérêt en Hémostase Périopératoire* (GIHP; working group on perioperative haemostasis) [115]	ICH, or haemorrhage in a critical organ	• Use of aPCC (30–50 IU/kg) or PCC (50 IU/kg) warranted, possibly re-administered once after an 8-h interval
	Emergent surgery	• PCCs should not be used for prophylactic reversal of anticoagulation, but PCC (25–50 IU/kg) or aPCC (30–50 IU/kg) should be considered to control perioperative bleeding events
*Groupe d’Intérêt en Hémostase Périopératoire* (GIHP; working group on perioperative haemostasis) [109]	Major intraoperative bleeding	• Multimodal approach, including 4F-PCC (25–50 IU/kg) or aPCC (30–50 IU/kg) administration
American Heart Association; American Stroke Association [78]	ICH	• aPCC or PCC may be considered on an individual basis
Neurocritical Care Society; Society of Critical Care Medicine [110]	ICH	• 4F-PCC (50 IU/kg) or aPCC (50 IU/kg) is suggested for FXa inhibitor-associated ICH • aPCC (50 IU/kg) or 4F-PCC (50 IU/kg) is suggested for dabigatran-associated ICH (if idarucizumab is not available)
Anticoagulation Forum [108]	Severe haemorrhage	• 4F-PCC (50 IU/kg) or aPCC (80 IU/kg) may be considered for reversal of direct FXa inhibitors and direct thrombin inhibitors, respectively
American Heart Association [116]	Serious bleeding or ICH	• 4F-PCC (50 IU/kg) is suggested for the treatment of patients receiving FXa inhibitors until more specific antidotes bcome available

*4F-PCC* four-factor prothrombin complex concentrate, *aPCC* activated prothrombin complex concentrate, *F* factor, *h* hour, *ICH* intracranial haemorrhage, *IU* international unit, *PCC* prothrombin complex concentrate

Perioperative management of patients receiving DOACs remains a challenge, particularly in cases of emergency surgery. A treatment algorithm proposed by the GIHP and other experts in the field suggests a multimodal approach to managing perioperative bleeding, which includes haemodynamic monitoring, standard resuscitation measures, and then ultimately PCC or aPCC administration [109].

Despite proposals recommending use of PCCs for treatment of DOAC-associated bleeding in certain clinical situations, one recent review suggested PCCs may not be useful for this purpose [126]. The author emphasises that addition of PCC usually does not correct the PT and may not completely normalise TGA parameters in the presence of FXa inhibitors. In addition, PCCs do not reduce the level of anti-Xa activity present in the plasma. The latter finding is expected, because PCCs do not directly inactivate FXa inhibitors as a true “reversal agent” would. It is important to note that laboratory parameters do not necessarily correlate well with clinical bleeding [19, 20, 26, 27, 32, 33]. Therefore, in cases where laboratory parameters are not fully normalised, a lack of clinically relevant benefit should not be assumed.

## Specific reversal agents

Specific reversal agents for DOACs are in development or have recently been licenced for DOAC-associated bleeding. The currently available agent, idarucizumab, is a monoclonal antibody fragment (Fab) that binds with high affinity to dabigatran. It was approved in the USA in October 2015 for dabigatran reversal in cases of major/uncontrolled bleeding or prior to emergency surgery/procedures [127]. This approval was based on data from studies in both healthy volunteers and patients requiring dabigatran reversal from the Reversal Effects of Idarucizumab on Active Dabigatran (RE-VERSE AD) cohort study [127, 128], which included a total of 503 patients presenting with serious bleeding (group A; *n* = 301) or requiring an urgent surgical or invasive procedure (group B; *n* = 202). dTT and ECT were normalised in 100% of patients in both groups A and group B, and the 30-day mortality rate was 13.5% in group A and 12.6% in group B, respectively.

Although idarucizumab is now licenced for dabigatran reversal, FXa inhibitors are more widely used than dabigatran [77]. The specific FXa inhibitor reversal agent Andexanet alfa (andexanet), a modified FXa mimetic that acts as a decoy and binds FXa inhibitors, has now received accelerated approval from the US Food and Drug Administration (FDA) for treatment of patients treated with rivaroxaban or apixaban who require reversal of the anticoagulant effects in life-threatening or uncontrolled bleeding. Although this agent reversed the anticoagulant effects of apixaban and rivaroxaban in a randomised controlled trial in healthy volunteers aged 50–75 years, data indicated that continuous infusions of andexanet are required to maintain this effect; anticoagulation reversal was maintained for the 2-h infusion period, with anti-Xa activity rebounding to placebo levels 1–3 h after the end of infusion [129]. The ETP increased above the normal range during the 2-h period, then declined after infusion was stopped. Of potential concern is that transient increases in D-dimer and prothrombin fragments 1 and 2 were observed in some patients, which may be related to the binding of andexanet to tissue factor pathway inhibitor [129]. In healthy volunteers, these elevations were not associated with thromboses; however, thrombotic events occurred in 12/67 (18%) patients included in a multicentre study evaluating andexanet in patients with acute major bleeding associated with the use of FXa inhibitors [130]. Patients (mean age, 77 years) presented predominantly with gastrointestinal or intracranial bleeding and were treated with an andexanet bolus (time from admission to therapy 4.8 ± 1.8 h), followed by a 2-h infusion. After bolus administration, median anti-factor Xa activity decreased by 89% and 93% in rivaroxaban- and apixaban-treated patients, respectively; these levels remained stable during the 2-h infusion, but partially returned to pre-treatment values shortly after. Clinical haemostasis 12 h after infusion was adjudicated as excellent or good in 37/47 (79%) patients included in the efficacy analysis, although it should be noted that TEEs occurred in 12 patients (18%).

Ciraparantag/aripazine (PER977), a small molecule that binds several anticoagulants, including DOACs and low molecular weight heparin, is in early phase 2 development [131]. A zymogen-like FXa variant is also in preclinical development [132].

Overall, more real-life data are needed for specific DOAC reversal agents, as there is some uncertainty about how they might perform in clinical practice. Agents have only been investigated in single-arm studies with no comparison group, making any effect on clinical haemostasis uncertain. Currently, there are no data in patients requiring FXa inhibitor reversal prior to emergency surgery.

## PCCs versus specific reversal agents

**Fig. 3:**
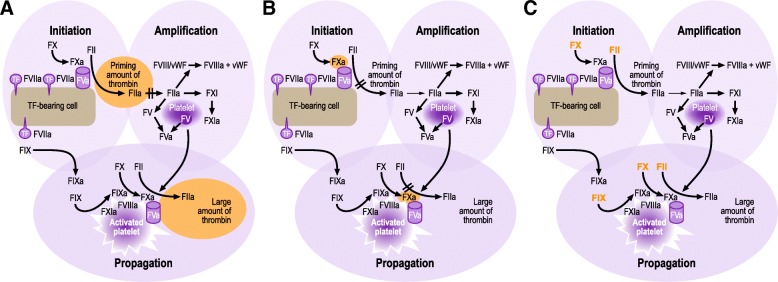
DOAC-induced anticoagulation and the proposed effect of PCCs. Figure legend: Direct inhibitors of FXa or thrombin (FIIa) affect both initiation and propagation of thrombin generation. FIIa inhibitors (e.g. dabigatran) directly inhibit the FIIa that is formed (**a**), while FXa inhibitors (e.g. rivaroxaban, edoxaban, apixaban) reduce the amount of thrombin generated by FXa (**b**). PCCs are thought to mitigate the anticoagulant effect of FXa or FIIa inhibitors, by increasing the levels of non-activated clotting factors (**c**), predominantly FII, FIX and FX, which can be activated in case of an active bleed. F, factor; FGN, fibrinogen; DOAC, non-vitamin K antagonist oral anticoagulant; PCC, prothrombin complex concentrate; TF, tissue factor; vWF, von Willebrand factor

As PCCs are the most commonly used DOAC reversal agents, it is important to compare their mechanism of action with those of the specific reversal agents. Specific reversal agents are enzymatically inactive and abrogate the effect of DOACs by direct binding, reducing the concentration of the anticoagulant in the blood. This effect can be measured by anti-Xa assays, in the case of FXa inhibitors, or the dTT, in the case of dabigatran. However, stoichiometric amounts of specific reversal agents (i.e. 1 molecule of antidote per molecule of anticoagulant) are needed to reverse the anticoagulant effect of DOACs (5 g of idarucizumab [127], 0.88–1.76 g of andexanet [129]). In contrast, PCCs are thought to mitigate the anticoagulant effect of DOACs by increasing the levels of non-activated clotting factors (Fig. 3), which can be activated in case of an active bleed. Therefore, small amounts of PCC can increase the potential to produce thrombin (which ultimately leads to the conversion of fibrinogen to fibrin and a stable clot). This suggests that administration of PCCs may be able to increase thrombin generation and reduce bleeding, without any effect on dTT or anti-Xa levels.

Although patient data on specific reversal agents are promising, the availability, cost and lack of data on these agents for surgical patients may impact widespread use [133]. The widespread use of PCCs for VKA reversal means that these agents are readily available in clinical practice.

In terms of safety, limited data are available regarding the incidence of thrombosis in patients receiving DOACs who are treated with PCCs; however, no such events occurred in the Dresden registry [65] or in a prospective observational study conducted in Japan [134]. A deep vein thrombosis was reported in a small chart review [74], and suspected or confirmed TEEs occurred in 3/84 patients treated with 4F-PCC (median dose 26.7 IU/kg) in the UPRATE study [67]. The authors note that this low rate (2.4%) is in line with that observed in VKA-treated patients or in cases where anticoagulation was discontinued without a reversal agent. TEEs were reported in 12/67 patients treated with andexanet for major bleeding associated with FXa inhibitors [130], and 5/90 patients treated with idarucizumab for dabigatran reversal due to serious bleeding or prior to urgent surgery [135]. Anticoagulant therapy had not been re-initiated in any of the cases in the idarucizumab study, which may suggest the occurrence of these events is due to underlying predisposing risk factors or a delay in re-initiating treatment, rather than the reversal agent used.

There have been no clinical studies directly comparing PCCs with specific antidotes; however, a preclinical study in a dabigatran-treated porcine polytrauma model showed that, as part of a multimodal therapy, high-dose 4F-PCC (50 IU/kg) was similarly effective to idarucizumab in reducing blood loss [38]. Furthermore, TEEs were not observed in either treatment group.

## Conclusion: PCCs as part of a clinical strategy for DOAC-associated bleeding

Despite a paucity of clinical data from patients with bleeding complications, an expanding body of evidence from preclinical models, healthy volunteers and clinical reports suggests PCCs have a potential role in the management of DOAC-associated bleeding. This is also reflected in a number of patient management guidance and guideline documents. Data are beginning to emerge from observational studies and large patient registries, which may assist the development of specific guidelines and strategies for DOAC reversal.

Although specific reversal agents for DOACs are available or in development, there may still be a role for PCCs, for example, in centres without access to these specific agents and in situations where the anticoagulant agent is unknown. Furthermore, once the anticoagulant effects of DOACs have been reversed using a specific reversal agent, patients may continue to bleed [133]. Concentrations of endogenous coagulation factors may be reduced due to loss during bleeding, consumption and dilution if patients are treated with fluids. Therefore, in cases where the bleeding event is particularly severe or prolonged, removing the anticoagulant effect may be insufficient to correct the coagulopathy. General consensus is that a multimodal approach should be used to restore haemostasis in patients presenting with DOAC-associated bleeding, and this may include the use of PCCs and other haemostatic agents.

## Additional files

Additional file 1: Search strategy and selection criteria. (DOCX 12 kb)

Additional file 2: Preclinical studies showing the effect of PCCs on bleeding in NOAC-treated animals (January 2013–February 2017). (DOCX 15 kb)

Additional file 3: Summary of ex vivo and in vitro studies showing the effect of PCCs on NOAC-induced coagulation (January 2013–February 2017). (DOCX 20 kb)

## Abbreviations

3F-PCC: Three-factor prothrombin complex concentrate
4F-PCC: Four-factor prothrombin complex concentrate
aPCC: Activated prothrombin complex concentrate
aPTT: Activated partial thromboplastin time
DOAC: Direct-acting oral anticoagulant
dTT: Diluted thrombin time
ECT: Ecarin clotting time
ETP: Endogenous thrombin potential
F: Factor
GIHP: *Groupe d’Intérêt en Hémostase Périopératoire* (French Working Group on Perioperative Haemostasis)
ICH: Intracranial haemorrhage
INR: International normalised ratio
ISTH: International Society on Thrombosis and Haemostasis
PCC: Prothrombin complex concentrate
PT: Prothrombin time
RASUNOA: Registry of Acute Stroke Under New Oral Anticoagulants
RE-VERSE AD: Reversal Effects of Idarucizumab on Active Dabigatran
ROTEM: Rotational thromboelastometry
TEE: Thromboembolic event
TEG: Thromboelastography
TGA: Thrombin generation assay
TT: Thrombin time
VKA: Vitamin K antagonist

## References

[1] Barnes GD, Lucas E, Alexander GC, Goldberger ZD (2015). National trends in ambulatory oral anticoagulant use. Am J Med.

[2] Ruff CT, Giugliano RP, Braunwald E, Hoffman EB, Deenadayalu N, Ezekowitz MD (2014). Comparison of the efficacy and safety of new oral anticoagulants with warfarin in patients with atrial fibrillation: a meta-analysis of randomised trials. Lancet.

[3] Abraham NS, Singh S, Alexander GC, Heien H, Haas LR, Crown W (2015). Comparative risk of gastrointestinal bleeding with dabigatran, rivaroxaban, and warfarin: population based cohort study. BMJ.

[4] Giugliano RP, Ruff CT, Braunwald E, Murphy SA, Wiviott SD, Halperin JL (2013). Edoxaban versus warfarin in patients with atrial fibrillation. N Engl J Med.

[5] Connolly SJ, Ezekowitz MD, Yusuf S, Eikelboom J, Oldgren J, Parekh A (2009). Dabigatran versus warfarin in patients with atrial fibrillation. N Engl J Med.

[6] Patel MR, Mahaffey KW, Garg J, Pan G, Singer DE, Hacke W (2011). Rivaroxaban versus warfarin in nonvalvular atrial fibrillation. N Engl J Med.

[7] Eikelboom J, Merli G (2016). Bleeding with direct oral anticoagulants vs warfarin: clinical experience. Am J Med.

[8] Dickneite G, Hoffman M (2014). Reversing the new oral anticoagulants with prothrombin complex concentrates (PCCs): what is the evidence?. Thromb Haemost.

[9] Eerenberg ES, Kamphuisen PW, Sijpkens MK, Meijers JC, Buller HR, Levi M (2011). Reversal of rivaroxaban and dabigatran by prothrombin complex concentrate: a randomized, placebo-controlled, crossover study in healthy subjects. Circulation.

[10] Baumann Kreuziger LM, Keenan JC, Morton CT, Dries DJ (2014). Management of the bleeding patient receiving new oral anticoagulants: a role for prothrombin complex concentrates. Biomed Res Int.

[11] Holbrook A, Schulman S, Witt DM, Vandvik PO, Fish J, Kovacs MJ (2012). Evidence-based management of anticoagulant therapy: antithrombotic therapy and prevention of thrombosis, 9th ed: American College of Chest Physicians Evidence-Based Clinical Practice Guidelines. Chest.

[12] Portola Pharmaceuticals (2018). U.S. FDA approves Portola Pharmaceuticals Andexxa®, the first and only antidote for the reversal of factor Xa inhibitors.

[13] Bosch YPJ, Weerwind PW, Mochtar B, Al DR (2014). Monitoring of haemostasis and anticoagulation in cardiopulmonary bypass patients. J Hematol Thrombo Dis.

[14] Curry ANG, Pierce TJM (2007). Conventional and near-patient tests of coagulation. Contin Educ Anaesth Crit Care Pain.

[15] Baglin T, Hillarp A, Tripodi A, Elalamy I, Buller H, Ageno W (2013). Measuring oral direct inhibitors (ODIs) of thrombin and factor Xa: a recommendation from the Subcommittee on Control of Anticoagulation of the Scientific and Standardisation Committee of the International Society on Thrombosis and Haemostasis. J Thromb Haemost.

[16] Ciurus T, Sobczak S, Cichocka-Radwan A, Lelonek M (2015). New oral anticoagulants - a practical guide. Kardiochir Torakochirurgia Pol.

[17] Lindahl TL, Baghaei F, Blixter IF, Gustafsson KM, Stigendal L, Sten-Linder M (2011). Effects of the oral, direct thrombin inhibitor dabigatran on five common coagulation assays. Thromb Haemost.

[18] Hawes EM, Deal AM, Funk-Adcock D, Gosselin R, Jeanneret C, Cook AM (2013). Performance of coagulation tests in patients on therapeutic doses of dabigatran: a cross-sectional pharmacodynamic study based on peak and trough plasma levels. J Thromb Haemost.

[19] Herzog E, Kaspereit F, Krege W, Mueller-Cohrs J, Doerr B, Niebl P (2015). Correlation of coagulation markers and 4F-PCC-mediated reversal of rivaroxaban in a rabbit model of acute bleeding. Thromb Res.

[20] Herzog E, Kaspereit F, Krege W, Mueller-Cohrs J, Doerr B, Niebl P (2015). Four-factor prothrombin complex concentrate reverses apixaban-associated bleeding in a rabbit model of acute hemorrhage. J Thromb Haemost.

[21] Escolar G, Fernandez-Gallego V, Arellano-Rodrigo E, Roquer J, Reverter JC, Sanz VV (2013). Reversal of apixaban induced alterations in hemostasis by different coagulation factor concentrates: significance of studies in vitro with circulating human blood. PLoS One.

[22] Frith D, Davenport R, Brohi K (2012). Acute traumatic coagulopathy. Curr Opin Anaesthesiol.

[23] Dias JD, Norem K, Doorneweerd DD, Thurer RL, Popovsky MA, Omert LA (2015). Use of thromboelastography (TEG) for detection of new oral anticoagulants. Arch Pathol Lab Med.

[24] Bliden K, Muresan A, Cohen E, Zaman F, Saadin K, Mohammed N (2015). Monitoring new oral anticoagulants (NOACS) using new-generation thrombelastography TEG®6S system. J Thromb Haemost.

[25] Artang R, Galloway G, Amiral J, Nielsen JD (2015). Monitoring novel anticoagulants dabigatran, rivaroxaban and apixaban using the new fully automated thrombelastography technique TEG®6S. J Thromb Haemost.

[26] Costin J, Ansell J, Laulicht B, Bakhru S, Steiner S (2014). Reversal agents in development for the new oral anticoagulants. Postgrad Med.

[27] Masotti L, Lorenzini G, Seravalle C, Panigada G, Landini G, Cappelli R (2015). Management of new oral anticoagulants related life threatening or major bleedings in real life: a brief report. J Thromb Thrombolysis.

[28] van Ryn J, Schurer J, Kink-Eiband M, Clemens A (2014). Reversal of dabigatran-induced bleeding by coagulation factor concentrates in a rat-tail bleeding model and lack of effect on assays of coagulation. Anesthesiology.

[29] Zhou W, Zorn M, Nawroth P, Butehorn U, Perzborn E, Heitmeier S (2013). Hemostatic therapy in experimental intracerebral hemorrhage associated with rivaroxaban. Stroke.

[30] Zahir H, Brown KS, Vandell AG, Desai M, Maa JF, Dishy V (2015). Edoxaban effects on bleeding following punch biopsy and reversal by a 4-factor prothrombin complex concentrate. Circulation.

[31] Herzog E, Kaspereit F, Krege W, Doerr B, Mueller-Cohrs J, Pragst I (2015). Effective reversal of edoxaban-associated bleeding with four-factor prothrombin complex concentrate in a rabbit model of acute hemorrhage. Anesthesiology.

[32] Herzog E, Kaspereit FJ, Krege W, Doerr B, van Ryn J, Dickneite G (2014). Thrombotic safety of prothrombin complex concentrate (Beriplex P/N) for dabigatran reversal in a rabbit model. Thromb Res.

[33] Hoffman M, Volovyk Z, Monroe DM (2015). Reversal of dabigatran effects in models of thrombin generation and hemostasis by factor VIIa and prothrombin complex concentrate. Anesthesiology.

[34] Honickel M, Braunschweig T, van Ryn J, Ten Cate H, Spronk HM, Rossaint R (2015). Prothrombin complex concentrate is effective in treating the anticoagulant effects of dabigatran in a porcine polytrauma model. Anesthesiology.

[35] Honickel M, Maron B, van Ryn J, Braunschweig T, Ten Cate H, Spronk HM, et al. Therapy with activated prothrombin complex concentrate is effective in reducing dabigatran-associated blood loss in a porcine polytrauma model. Thromb Haemostasis. 2016;115(2):271–84. 10.1160/TH15-03-0266 26333775

[36] Martin AC, Le Bonniec B, Fischer AM, Marchand-Leroux C, Gaussem P, Samama CM (2013). Evaluation of recombinant activated factor VII, prothrombin complex concentrate, and fibrinogen concentrate to reverse apixaban in a rabbit model of bleeding and thrombosis. Int J Cardiol.

[37] Perzborn E, Gruber A, Tinel H, Marzec UM, Buetehorn U, Buchmueller A (2013). Reversal of rivaroxaban anticoagulation by haemostatic agents in rats and primates. Thromb Haemost.

[38] Honickel M, Braunschweig T, Rossaint R, Stoppe C, Ten Cate H, Grottke O (2017). Reversing dabigatran anticoagulation with prothrombin complex concentrate versus idarucizumab as part of multimodal hemostatic intervention in an animal model of polytrauma. Anesthesiology.

[39] Arellano-Rodrigo E, Lopez-Vilchez I, Galan AM, Molina P, Reverter JC, Carne X (2015). Coagulation factor concentrates fail to restore alterations in fibrin formation caused by rivaroxaban or dabigatran in studies with flowing blood from treated healthy volunteers. Transfus Med Rev.

[40] Dinkelaar J, Molenaar PJ, Ninivaggi M, de Laat B, Brinkman HJ, Leyte A (2013). In vitro assessment, using thrombin generation, of the applicability of prothrombin complex concentrate as an antidote for rivaroxaban. J Thromb Haemost.

[41] Dinkelaar J, Patiwael S, Harenberg J, Leyte A, Brinkman HJ (2014). Global coagulation tests: their applicability for measuring direct factor Xa- and thrombin inhibition and reversal of anticoagulation by prothrombin complex concentrate. Clin Chem Lab Med.

[42] Escolar G, Arellano-Rodrigo E, Lopez-Vilchez I, Molina P, Sanchis J, Reverter JC (2015). Reversal of rivaroxaban-induced alterations on hemostasis by different coagulation factor concentrates - in vitro studies with steady and circulating human blood. Circ J.

[43] Halim AB, Samama MM, Mendell J (2014). Ex vivo reversal of the anticoagulant effects of edoxaban. Thromb Res.

[44] Herrmann R, Thom J, Wood A, Phillips M, Muhammad S, Baker R (2014). Thrombin generation using the calibrated automated thrombinoscope to assess reversibility of dabigatran and rivaroxaban. Thromb Haemost.

[45] Iba T, Emmi M, Hiki M, Nagayama M, Aihara K, Tabe Y (2016). Comparison of prothrombin time tests used in the monitoring of edoxaban and their evaluation as indicators of the reversal effect. Int J Hematol.

[46] Khoo TL, Weatherburn C, Kershaw G, Reddel CJ, Curnow J, Dunkley S (2013). The use of FEIBA(R) in the correction of coagulation abnormalities induced by dabigatran. Int J Lab Hematol.

[47] Korber MK, Langer E, Kaufner L, Sander M, Von Heymann C (2016). In vitro reversal of supratherapeutic rivaroxaban levels with coagulation factor concentrates. Blood Transfus.

[48] Körber MK, Langer E, Ziemer S, Perzborn E, Gericke C, Heymann C (2014). Measurement and reversal of prophylactic and therapeutic peak levels of rivaroxaban: an in vitro study. Clin Appl Thromb Hemost.

[49] Lindahl TL, Wallstedt M, Gustafsson KM, Persson E, Hillarp A (2015). More efficient reversal of dabigatran inhibition of coagulation by activated prothrombin complex concentrate or recombinant factor VIIa than by four-factor prothrombin complex concentrate. Thromb Res.

[50] Martin AC, Gouin-Thibault I, Siguret V, Mordohay A, Samama CM, Gaussem P (2015). Multimodal assessment of non-specific hemostatic agents for apixaban reversal. J Thromb Haemost.

[51] Perzborn E, Heitmeier S, Laux V, Buchmuller A (2014). Reversal of rivaroxaban-induced anticoagulation with prothrombin complex concentrate, activated prothrombin complex concentrate and recombinant activated factor VII in vitro. Thromb Res.

[52] Schenk B, Wurtinger P, Streif W, Sturm W, Fries D, Bachler M (2016). Ex vivo reversal of effects of rivaroxaban evaluated using thromboelastometry and thrombin generation assay. Br J Anaesth.

[53] Schultz NH, Tran HT, Bjornsen S, Henriksson CE, Sandset PM, Holme PA (2017). The reversal effect of prothrombin complex concentrate (PCC), activated PCC and recombinant activated factor VII against anticoagulation of Xa inhibitor. Thromb J.

[54] Solbeck S, Meyer MA, Johansson PI, Meyer AS, Cotton BA, Stensballe J (2014). Monitoring of dabigatran anticoagulation and its reversal in vitro by thrombelastography. Int J Cardiol.

[55] Solbeck S, Nilsson CU, Engstrom M, Ostrowski SR, Johansson PI (2014). Dabigatran and its reversal with recombinant factor VIIa and prothrombin complex concentrate: a Sonoclot in vitro study. Scand J Clin Lab Invest.

[56] Nagakari K, Emmi M, Iba T (2017). Prothrombin time tests for the monitoring of direct oral anticoagulants and their evaluation as indicators of the reversal effect. Clin Appl Thromb Hemost.

[57] Levi M, Moore KT, Castillejos CF, Kubitza D, Berkowitz SD, Goldhaber SZ (2014). Comparison of three-factor and four-factor prothrombin complex concentrates regarding reversal of the anticoagulant effects of rivaroxaban in healthy volunteers. J Thromb Haemost.

[58] Barco S, Whitney Cheung Y, Coppens M, Hutten BA, Meijers JC, Middeldorp S (2015). In vivo reversal of the anticoagulant effect of rivaroxaban with four-factor prothrombin complex concentrate. Br J Haematol.

[59] Levy JH, Moore KT, Neal MD, Schneider D, Marcsisin VS, Ariyawansa J (2018). Rivaroxaban reversal with prothrombin complex concentrate or tranexamic acid in healthy volunteers. J Thromb Haemost.

[60] Brown KS, Wickremasingha P, Parasrampuria DA, Weiss D, Kochan J, Dishy V (2015). The impact of a three-factor prothrombin complex concentrate on the anticoagulatory effects of the factor Xa inhibitor edoxaban. Thromb Res.

[61] Cheung YW, Barco S, Hutten BA, Meijers JC, Middeldorp S, Coppens M (2015). In vivo increase in thrombin generation by four-factor prothrombin complex concentrate in apixaban-treated healthy volunteers. J Thromb Haemost.

[62] Song Y, Wang Z, Perlstein I, Wang J, LaCreta F, Frost RJA (2017). Reversal of apixaban anticoagulation by 4-factor prothrombin complex concentrates in healthy subjects: a randomized 3-period crossover study. J Thromb Haemost.

[63] Nagalla S, Thomson L, Oppong Y, Bachman B, Chervoneva I, Kraft WK (2016). Reversibility of apixaban anticoagulation with a four-factor prothrombin complex concentrate in healthy volunteers. Clin Transl Sci.

[64] Schulman S, Kearon C (2005). Subcommittee on Control of Anticoagulation of the S, Standardization Committee of the International Society on T, Haemostasis. Definition of major bleeding in clinical investigations of antihemostatic medicinal products in non-surgical patients. J Thromb Haemost.

[65] Beyer-Westendorf J, Forster K, Pannach S, Ebertz F, Gelbricht V, Thieme C (2014). Rates, management, and outcome of rivaroxaban bleeding in daily care: results from the Dresden NOAC registry. Blood.

[66] Albaladejo P, Samama CM, Sie P, Kauffmann S, Memier V, Suchon P (2017). Management of severe bleeding in patients treated with direct oral anticoagulants: an observational registry analysis. Anesthesiology.

[67] Majeed A, Agren A, Holmstrom M, Bruzelius M, Chaireti R, Odeberg J (2017). Management of rivaroxaban or apixaban associated major bleeding with prothrombin complex concentrates: a cohort study. Blood.

[68] Schulman S, Gross PL, Ritchie B, Nahirniak S, Lin Y, Lieberman L (2018). Prothrombin complex concentrate for major bleeding on factor Xa inhibitors: a prospective cohort study. Thromb Haemost.

[69] Schulman S, Ritchie B, Nahirniak S, Gross PL, Carrier M, Majeed A (2017). Reversal of dabigatran-associated major bleeding with activated prothrombin concentrate: a prospective cohort study. Thromb Res.

[70] Lindhoff-Last E (2017). Direct oral anticoagulants (DOAC) - management of emergency situations. Rationale and design of the RADOA-Registry. Hamostaseologie.

[71] Schenk B, Goerke S, Beer R, Helbok R, Fries D, Bachler M (2018). Four-factor prothrombin complex concentrate improves thrombin generation and prothrombin time in patients with bleeding complications related to rivaroxaban: a single-center pilot trial. Thromb J.

[72] Xu Y, Schulman S, Dowlatshahi D, Holbrook AM, Simpson CS, Shepherd LE (2017). Direct oral anticoagulant- or warfarin-related major bleeding: characteristics, reversal strategies and outcomes from a multi-center observational study. Chest.

[73] Pahs L, Beavers C, Schuler P (2015). The real-world treatment of hemorrhages associated with dabigatran and rivaroxaban: a multicenter evaluation. Crit Pathw Cardiol.

[74] Tellor KB, Barasch NS, Lee BM. Clinical experience reversing factor Xa inhibitors with four-factor prothrombin complex concentrate in a community hospital. Blood Transfus. 2018;16(4):382–86. 10.2450/2017.0219-16 PMC6034772 28151386

[75] Aguilar MI, Brott TG (2011). Update in intracerebral hemorrhage. The Neurohospitalist.

[76] Purrucker JC, Haas K, Rizos T, Khan S, Wolf M, Hennerici MG, et al. Early clinical and radiological course, management, and outcome of intracerebral hemorrhage related to new oral anticoagulants. JAMA Neurol. 2016;73(2):169–77. 10.1001/jamaneurol.2015.3682 26660118

[77] IMS Health (2015). Prescription audit.

[78] Hemphill JC, Greenberg SM, Anderson CS, Becker K, Bendok BR, Cushman M (2015). Guidelines for the management of spontaneous intracerebral hemorrhage: a guideline for healthcare professionals from the American Heart Association/American Stroke Association. Stroke.

[79] Gerner ST, Kuramatsu JB, Sembill JA, Sprugel MI, Endres M, Haeusler KG (2018). Association of prothrombin complex concentrate administration and hematoma enlargement in non-vitamin K antagonist oral anticoagulant-related intracerebral hemorrhage. Ann Neurol.

[80] Beynon C, Sakowitz OW, Storzinger D, Orakcioglu B, Radbruch A, Potzy A (2015). Intracranial haemorrhage in patients treated with direct oral anticoagulants. Thromb Res.

[81] Kuramatsu JB, Gerner ST, Schellinger PD, Glahn J, Endres M, Sobesky J (2015). Anticoagulant reversal, blood pressure levels, and anticoagulant resumption in patients with anticoagulation-related intracerebral hemorrhage. JAMA.

[82] Grandhi R, Newman WC, Zhang X, Harrison G, Moran C, Okonkwo DO (2015). Administration of 4-factor prothrombin complex concentrate as an antidote for intracranial bleeding in patients taking direct factor Xa inhibitors. World Neurosurg.

[83] Mao G, King L, Young S, Kaplan R (2017). Factor eight inhibitor bypassing agent (FEIBA) for reversal of target-specific oral anticoagulants in life-threatening intracranial bleeding. J Emerg Med.

[84] Yin EB, Tan B, Nguyen T, Salazar M, Putney K, Gupta P (2017). Safety and effectiveness of factor VIII inhibitor bypassing activity (FEIBA) and fresh frozen plasma in oral anticoagulant-associated intracranial hemorrhage: a retrospective analysis. Neurocrit Care.

[85] Gianni C, Di Biase L, Mohanty S, Trivedi C, Bai R, Al-Ahmad A (2016). Management of periprocedural and early pericardial effusions with tamponade following ablation of atrial fibrillation with uninterrupted factor Xa inhibitors: a case series. J Cardiovasc Electrophysiol.

[86] Dibu JR, Weimer JM, Ahrens C, Manno E, Frontera JA (2016). The role of FEIBA in reversing novel oral anticoagulants in intracerebral hemorrhage. Neurocrit Care.

[87] Faust AC, Peterson EJ (2014). Management of dabigatran-associated intracerebral and intraventricular hemorrhage: a case report. J Emerg Med.

[88] Rinehart DR, Lockhart NR, Hamilton LA, Langdon JR, Rowe AS (2015). Management of apixaban-associated subdural hematoma: a case report on the use of factor eight inhibitor bypassing activity. Crit Care Med.

[89] Beynon C, Potzy A, Unterberg AW, Sakowitz OW (2015). Emergency neurosurgical care in patients treated with apixaban: report of 2 cases. Am J Emerg Med.

[90] Chic Acevedo C, Velasco F, Herrera C (2015). Reversal of rivaroxaban anticoagulation by nonactivated prothrombin complex concentrate in urgent surgery. Futur Cardiol.

[91] Maurice-Szamburski A, Graillon T, Bruder N (2014). Favorable outcome after a subdural hematoma treated with feiba in a 77-year-old patient treated by rivaroxaban. J Neurosurg Anesthesiol.

[92] Neyens R, Bohm N, Cearley M, Andrews C, Chalela J (2014). Dabigatran-associated subdural hemorrhage: using thromboelastography (TEG((R))) to guide decision-making. J Thromb Thrombolysis.

[93] Puttick T, Bahl R, Mohamedbhai H. Emergency reversal of dabigatran for emergency surgery. BMJ Case Rep. 2015. 10.1136/bcr-2014-209057 10.1136/bcr-2014-209057 PMC4422923 25926585

[94] Wong H, Keeling D (2014). Activated prothrombin complex concentrate for the prevention of dabigatran-associated bleeding. Br J Haematol.

[95] Dager WE, Gosselin RC, Roberts AJ (2013). Reversing dabigatran in life-threatening bleeding occurring during cardiac ablation with factor eight inhibitor bypassing activity. Crit Care Med.

[96] Denetclaw TH, Tam J, Arias V, Kim R, Martin C (2016). Case report: apixaban-associated gluteal artery extravasation reversed with PCC3 without FFP. J Pharm Pract.

[97] Diaz MQ, Borobia AM, Nunez MA, Virto AM, Fabra S, Casado MS (2013). Use of prothrombin complex concentrates for urgent reversal of dabigatran in the emergency department. Haematologica.

[98] Kauffmann S, Chabanne R, Coste A, Longeras F, Sinegre T, Schmidt J (2015). Favorable outcome of rivaroxaban-associated intracerebral hemorrhage reversed by 4-factor prothrombin complex concentrate: impact on thrombin generation. A A Case Rep.

[99] McGovern TR, McNamee JJ, Malabanan C, Fouad MA, Patel N (2015). Use of 4-factor prothrombin complex concentrate in the treatment of a gastrointestinal hemorrhage complicated by dabigatran. Int J Emerg Med.

[100] Schulman S, Ritchie B, Goy JK, Nahirniak S, Almutawa M, Ghanny S (2014). Activated prothrombin complex concentrate for dabigatran-associated bleeding. Br J Haematol.

[101] Durie R, Kohute M, Fernandez C, Knight M (2016). Prothrombin complex concentrate for the management of severe traumatic bleeding in a patient anticoagulated with apixaban. J Clin Pharm Ther.

[102] Faust AC, Woodard S, Koehl JL, Mees W, Steinke D, Denetclaw TH (2016). Managing subdural bleeding associated with rivaroxaban: a series of 3 cases. J Pharm Pract.

[103] Means L, Gimbar RP (2016). Prothrombin complex concentrate administration through intraosseous access for reversal of rivaroxaban. Am J Emerg Med.

[104] Jones JM, Ryan HM, Tieszen M, Leedahl DD (2016). Successful hemostasis and reversal of highly elevated PT/INR after dabigatran etexilate use in a patient with acute kidney injury. Am J Emerg Med.

[105] Liu M, Aguele C, Darr U (2017). Use of four-factor prothrombin complex concentrate for the mitigation of rivaroxaban-induced bleeding in an emergent coronary artery bypass graft. Clin Case Rep.

[106] Smith AT, Wrenn KD, Barrett TW, Jones ID, Rohde JP, Slovis CM (2017). Delayed intracranial hemorrhage after head trauma in patients on direct-acting oral anticoagulants. Am J Emerg Med.

[107] Faust AC, Tran DM, Lo C, Lai S, Sheperd L, Liu M (2018). Managing nonoperable intracranial bleeding associated with apixaban: a series of 2 cases. J Pharm Pract.

[108] Burnett AE, Mahan CE, Vazquez SR, Oertel LB, Garcia DA, Ansell J (2016). Guidance for the practical management of the direct oral anticoagulants (DOACs) in VTE treatment. J Thromb Thrombolysis.

[109] Faraoni D, Levy JH, Albaladejo P, Samama CM (2015). Updates in the perioperative and emergency management of non-vitamin K antagonist oral anticoagulants. Crit Care.

[110] Frontera JA, Lewin JJ, Rabinstein AA, Aisiku IP, Alexandrov AW, Cook AM (2016). Guideline for reversal of antithrombotics in intracranial hemorrhage : a statement for healthcare professionals from the Neurocritical Care Society and Society of Critical Care Medicine. Neurocrit Care.

[111] Heidbuchel H, Verhamme P, Alings M, Antz M, Diener HC, Hacke W (2015). Updated European heart rhythm association practical guide on the use of non-vitamin K antagonist anticoagulants in patients with non-valvular atrial fibrillation. Europace.

[112] Heidbuchel H, Verhamme P, Alings M, Antz M, Hacke W, Oldgren J (2013). European Heart Rhythm Association Practical Guide on the use of new oral anticoagulants in patients with non-valvular atrial fibrillation. Europace.

[113] Kaatz S, Kouides PA, Garcia DA, Spyropolous AC, Crowther M, Douketis JD (2012). Guidance on the emergent reversal of oral thrombin and factor Xa inhibitors. Am J Hematol.

[114] Kozek-Langenecker SA, Afshari A, Albaladejo P, Santullano CA, De Robertis E, Filipescu DC (2013). Management of severe perioperative bleeding: guidelines from the European Society of Anaesthesiology. Eur J Anaesthesiol.

[115] Pernod G, Albaladejo P, Godier A, Samama CM, Susen S, Gruel Y (2013). Management of major bleeding complications and emergency surgery in patients on long-term treatment with direct oral anticoagulants, thrombin or factor-Xa inhibitors: proposals of the working group on perioperative haemostasis (GIHP) - March 2013. Arch Cardiovasc Dis.

[116] Raval AN, Cigarroa JE, Chung MK, Diaz-Sandoval LJ, Diercks D, Piccini JP (2017). Management of patients on non-vitamin K antagonist oral anticoagulants in the acute care and periprocedural setting: a scientific statement from the American Heart Association. Circulation.

[117] Spahn DR, Bouillon B, Cerny V, Coats TJ, Duranteau J, Fernandez-Mondejar E (2013). Management of bleeding and coagulopathy following major trauma: an updated European guideline. Crit Care.

[118] Tran HA, Chunilal SD, Harper PL, Tran H, Wood EM, Gallus AS (2013). An update of consensus guidelines for warfarin reversal. Med J Aust.

[119] Peacock WF, Gearhart MM, Mills RM (2012). Emergency management of bleeding associated with old and new oral anticoagulants. Clin Cardiol.

[120] Fawole A, Daw HA, Crowther MA (2013). Practical management of bleeding due to the anticoagulants dabigatran, rivaroxaban, and apixaban. Cleve Clin J Med.

[121] Turpie AG, Kreutz R, Llau J, Norrving B, Haas S (2012). Management consensus guidance for the use of rivaroxaban--an oral, direct factor Xa inhibitor. Thromb Haemost.

[122] Levy JH, Faraoni D, Spring JL, Douketis JD, Samama CM (2013). Managing new oral anticoagulants in the perioperative and intensive care unit setting. Anesthesiology.

[123] FDA. XARELTO® (rivaroxaban) Prescribing Information 2015 [Available from: https://www.xareltohcp.com/shared/product/xarelto/prescribing-information.pdf.

[124] FDA. ELIQUIS® (apixaban) Prescribing Information 2015 [Available from: http://packageinserts.bms.com/pi/pi_eliquis.pdf.

[125] de Schipper LJ, Baharoglu MI, Roos Y, de Beer F (2017). Medical treatment for spontaneous anticoagulation-related intracerebral hemorrhage in the Netherlands. J Stroke Cerebrovasc Dis.

[126] Dzik WH (2015). Reversal of oral factor Xa inhibitors by prothrombin complex concentrates: a re-appraisal. J Thromb Haemost.

[127] Boehringer Ingelheim. Praxbind® Prescribing Information 2015 [Available from: http://docs.boehringer-ingelheim.com/Prescribing%20Information/PIs/Praxbind/Praxbind.pdf?DMW_FORMAT=pdf.

[128] Pollack CV, Reilly PA, van Ryn J, Eikelboom JW, Glund S, Bernstein RA (2017). Idarucizumab for dabigatran reversal - full cohort analysis. N Engl J Med.

[129] Siegal DM, Curnutte JT, Connolly SJ, Lu G, Conley PB, Wiens BL (2015). Andexanet alfa for the reversal of factor Xa inhibitor activity. N Engl J Med.

[130] Connolly SJ, Milling TJ, Eikelboom JW, Gibson CM, Curnutte JT, Gold A (2016). Andexanet alfa for acute major bleeding associated with factor Xa inhibitors. N Engl J Med.

[131] Ansell JE, Bakhru SH, Laulicht BE, Steiner SS, Grosso M, Brown K (2014). Use of PER977 to reverse the anticoagulant effect of edoxaban. N Engl J Med.

[132] Thalji NK, Ivanciu L, Davidson R, Gimotty PA, Krishnaswamy S, Camire RM (2016). A rapid pro-hemostatic approach to overcome direct oral anticoagulants. Nat Med.

[133] Levy JH, Ageno W, Chan NC, Crowther M, Verhamme P, Weitz JI (2016). When and how to use antidotes for the reversal of direct oral anticoagulants: guidance from the SSC of the ISTH. J Thromb Haemost.

[134] Yoshimura S, Sato S, Todo K, Okada Y, Furui E, Matsuki T (2017). Prothrombin complex concentrate administration for bleeding associated with non-vitamin K antagonist oral anticoagulants: the SAMURAI-NVAF study. J Neurol Sci.

[135] Pollack CV, Reilly PA, Eikelboom J, Glund S, Verhamme P, Bernstein RA (2015). Idarucizumab for dabigatran reversal. N Engl J Med.

[136] Granger CB, Alexander JH, McMurray JJ, Lopes RD, Hylek EM, Hanna M (2011). Apixaban versus warfarin in patients with atrial fibrillation. N Engl J Med.

