# Supplementary material for: The impact of prothrombin complex concentrates when treating DOAC-associated bleeding: a review
Source: Int J Emerg Med. 2018 Dec 3;11:55. doi: 10.1186/s12245-018-0215-6 (PMC6326120; doi:10.1186/s12245-018-0215-6)
Supplement: Supplementary file 1 — Search strategy and selection criteria. (DOCX 12 kb) [file 12245_2018_215_MOESM1_ESM.docx]

Additional File 1. Search strategy and selection criteria

Papers were identified by PubMed searches using the following search terms: (prothrombin complex concentrate OR prothrombin complex concentrates OR beriplex OR octaplex OR PPSB OR kaskadil OR cofact OR bebulin OR profilnine OR prothrombinex OR FEIBA) AND (oral anticoagulant OR apixaban OR rivaroxaban OR edoxaban OR dabigatran).

Relevant articles published in English from January 2013 to February 2018 were include
