# Supplementary material for: The impact of prothrombin complex concentrates when treating DOAC-associated bleeding: a review
Source: Int J Emerg Med. 2018 Dec 3;11:55. doi: 10.1186/s12245-018-0215-6 (PMC6326120; doi:10.1186/s12245-018-0215-6)
Supplement: Supplementary file 2 — Preclinical studies showing the effect of PCCs on bleeding in NOAC-treated animals (January 2013–February 2017). (DOCX 15 kb) [file 12245_2018_215_MOESM2_ESM.docx]

### Additional File 2. Preclinical studies showing the effect of PCCs on bleeding in NOAC-treated animals (January 2013–February 2017)

| Study citation | PCC | Model | Results |
| --- | --- | --- | --- |
| Dabigatran | | | |
| Hoffman *et al*. 2015 | 4F-PCC (Beriplex®) | Mouse, saphenous vein bleeding model | ↓ time to haemostasis |
| Honickel *et al*. 2015 and Honickel *et al.* 2017 | 4F-PCC (Beriplex®) | Pig, polytrauma model | ↓ blood loss |
| Herzog *et al*. 2014 | 4F-PCC (Beriplex®) | Rabbit, kidney incision model (and arterial venous shunt) | ↓ blood loss and time to haemostasis |
| van Ryn *et al*. 2014 | 3F-PCCs (Profilnine® and Bebulin®), 4F-PCCs (Beriplex® and Octaplex®) and aPCC (FEIBA®) | Rat, tail incision model | ↓ blood loss |
| Honickel *et al*. 2015 | aPCC (FEIBA®) | Pig, polytrauma model | ↓ blood loss |
| Rivaroxaban | | | |
| Herzog *et al*. 2015 | 4F-PCC (Beriplex ®) | Rabbit, kidney incision model | ↓ blood loss and time to haemostasis |
| Zhou *et al*. 2013 | 4F-PCC (Beriplex ®) | Mouse, ICH model | ↓ haematoma volume |
| Perzborn *et al*. 2013 | 4F-PCC (Beriplex®) and aPCC (FEIBA®) | Rat, mesenteric bleeding model | ↓ bleeding time |
|  | aPCC (FEIBA®) | Baboon, mesenteric bleeding model | ↓ bleeding time (not sustained after end of infusion) |
| Edoxaban | | | |
| Herzog *et al*. 2015 | 4F-PCC (Beriplex®) | Rabbit, kidney incision model | ↓ blood loss and time to haemostasis |
| Apixaban | | | |
| Herzog *et al*. 2015 | 4F-PCC (Beriplex®) | Rabbit, kidney incision model | ↓ blood loss and time to haemostasis |
| Martin *et al*. 2013 | 4F-PCC (Kanokad®) | Rabbit, hepatosplenic bleeding model | No reduction in blood loss or bleeding time |

↓, reduced.

3F-PCC, three-factor prothrombin complex concentrate; 4F-PCC, four-factor prothrombin complex concentrate; aPCC, activated prothrombin complex concentrate; FEIBA, factor eight inhibitor bypassing activity; ICH, intracranial haemorrhage.
