# Supplementary material for: The impact of prothrombin complex concentrates when treating DOAC-associated bleeding: a review
Source: Int J Emerg Med. 2018 Dec 3;11:55. doi: 10.1186/s12245-018-0215-6 (PMC6326120; doi:10.1186/s12245-018-0215-6)
Supplement: Supplementary file 3 — Summary of ex vivo and in vitro studies showing the effect of PCCs on NOAC-induced coagulation (January 2013–February 2017). (DOCX 20 kb) [file 12245_2018_215_MOESM3_ESM.docx]

### Additional File 3. Summary of *ex vivo* and *in vitro* studies showing the effect of PCCs on NOAC-induced coagulation (January 2013–February 2017)

| Study citation | NOAC | PCC | Laboratory parameters tested | Reversal of NOAC effects with PCC |
| --- | --- | --- | --- | --- |
| *ex vivo* studies using samples from NOAC-treated patients | | | | |
| Khoo *et al*. 2013 | Dabigatran | aPCC (FEIBA®) | TGA parameters: peak TG, lag time, ETP | (+) |
| Herrmann *et al*. 2014 | Dabigatran | 3F‑PCC  (Prothrombinex®) or aPCC (FEIBA®) | aPTT and TCT  PT  CAT parameters: lag time, ETP and peak | (+) with both PCCs  (+) with aPCC only  (+) with both PCCs |
|  | Rivaroxaban |  | PT  aPTT  TCT  CAT parameters: lag time, ETP and peak | (+) with both PCCs  (+) with aPCC only  (+) with 3F-PCC only  (+) with both PCCs |
| Schenk *et al*. 2016 | Rivaroxaban | 4F-PCC (Beriplex®) or aPCC (FEIBA®) | TEM parameters: CT  TGA parameters:   - ETP - Peak - Lag time | (+) with both PCCs, aPCC > 4F-PCC (reagent dependent)  (+) with both PCCs, aPCC > 4F-PCC  (+) with both PCCs, 4F-PCC > aPCC  (+) with both PCCs, aPCC > 4F-PCC |
| Schultz *et al*. 2017 | Rivaroxaban | 4F-PCC (Cofact®) or aPCC (FEIBA®) | TGA parameters: lag time, peak, velocity index, ETP  TEM parameters: CT, CFT, MCF, maximum velocity, AUC | (+) with both PCCs, aPCC > 4F-PCC (no significant effect on lag time with PCC)  (+) with both PCCs, aPCC > 4F-PCC (no effect on MCF, maximum velocity and AUC) |
| *ex vivo* study using samples from NOAC-dosed healthy volunteers | | | | |
| Arellano-Rodrigo *et al*. 2015 | Dabigatran | 4F‑PCC (Beriplex®) or aPCC (FEIBA®) | PT  aPTT  TGA parameters: lag time, ETP, peak, time to peak  TEM parameters: CT, CFT and MCF | Partial with both PCCs  (+) with aPCC only  (+) with both PCCs  (+) with aPCC only *(no effect on CFT)* |
|  | Rivaroxaban |  | PT  aPTT  TGA parameters: lag time, ETP, peak, time to peak  TEM parameters: CT, CFT and MCF | (+) with aPCC; partial with 4F-PCC  (+) with aPCC only  (+) with aPCC; lag time and ETP only with 4F-PCC  (+) with both PCCs |
| *in vitro* studies using samples from healthy volunteers | | | | |
| Solbeck *et al*. 2014a | Dabigatran | 4F-PCC (Octaplex®) | TEG parameters | (‒) |
| Solbeck *et al*. 2014b | Dabigatran | 4F-PCC (Octaplex®) | Sonoclot parameters | (‒) |
| Lindahl *et al*. 2015 | Dabigatran | 4F-PCC (Cofact®) or aPCC (FEIBA®) | CT  Initial rate of clot growth | (+) with aPCC only  (‒) |
| Perzborn *et al*. 2014 | Rivaroxaban | 4F-PCC (Beriplex®) or aPCC (FEIBA®) | PT  CT  TGA parameters:   - ETP - Lag time - Peak | (+) with both PCCs, aPCC > 4F-PCC  Partial with aPCC only  (+) with both PCCs, aPCC > 4F-PCC  Partial with both PCCs, aPCC > 4F-PCC  Partial with aPCC only |
| Escolar *et al*. 2015 | Rivaroxaban | 4F-PCC (Beriplex®) or aPCC (FEIBA®) | TGA parameters  TEM parameters: CT, CFT and MCF | (+) with both PCCs, aPCC > 4F-PCC  (+) with both PCCs, aPCC > 4F-PCC |
| Körber *et al*. 2014 | Rivaroxaban | 4F-PCC (Octaplex®) | CT, aPTT, PT ratio, PT | (‒) |
| Körber *et al*. 2016 | Rivaroxaban | 4F-PCC (Octaplex®) or aPCC (FEIBA®) | CT  aPTT  PT ratio  PT | (+) (significant effect dose-dependent with 4F-PCC)  (+)  (+) with aPCC only  (+) with aPCC only |
| Dinkelaar *et al*. 2013 | Rivaroxaban | 4F-PCC (Cofact®) | ETP, peak  PT, lag time | (+) (in whole blood CAT)  (‒) |
| Nagakari *et al*. 2017 | Rivaroxaban, edoxaban or apixaban | 4F-PCC (PPSB-HT®) or aPCC | PT | (+) |
| Iba *et al*. 2016 | Edoxaban | 4F-PCC (PPSB-HT®) or aPCC | PT | (+) |
| Halim *et al*. 2014 | Edoxaban | aPCC (FEIBA®) | PT and aPTT | (+) |
| Escolar *et al*. 2013 | Apixaban | 4F-PCC (Beriplex®) or aPCC (FEIBA®) | TGA parameters:   - Lag phase, peak (phospholipid reagent) - Time to peak, peak (TF and phospholipid reagent)   TEM parameters: CT, CFT, MCF | (+) with both PCCs  (+) with aPCC only  (+) with aPCC; significant effect only observed for MCF with 4F-PCC |
| Martin *et al*. 2015 | Apixaban | 4F-PCC (Kanokad®) or aPCC (FEIBA®) | TGA parameters   - ETP - Lag time - Peak height   PT  aPTT  Fibrin polymerisation kinetics  TEM parameters | (+) with both PCCs, aPCC > 4F-PCC  (+) with 4F-PCC, overcorrection with aPCC  (+) with aPCC only  (+) with both PCCs  (+) with both PCCs  Partial with aPCC only  (+) with aPCC only  Partial with aPCC only *(no effect on MCF)* |
| Dinkelaar *et al*. 2014 | Apixaban | 4F-PCC (Cofact®) | PT  ETP  TEG parameters  aPTT, peak, lag time | (+)  (+)  Partial for pooled plasma (no effect on whole blood TEG parameters)  (‒) |
|  | Dabigatran |  | PT  ETP  Peak TG  Whole blood TEG parameters  aPTT, lag time | Partial  (+)  (+)  (+) dependent on reagent used for whole blood *(no effect on pooled plasma TEG parameters)*  (‒) |

(‒), negative result; (+), positive result.

3F-PCC, three-factor prothrombin complex concentrate; 4F-PCC, four-factor prothrombin complex concentrate; aPCC, activated prothrombin complex concentrate; aPTT, activated partial thromboplastin time; AUC, area under the curve; CAT, calibrated automated thrombogram; CFT, clot formation time; CT, clotting time; ETP, endogenous thrombin potential; MCF, maximum clot firmness; PT, prothrombin time; TCT, thrombin clotting time; TEG, thromboelastography; TF, tissue factor; TG, thrombin generation; TGA, thrombin generation assay; TT, thrombin time; TEM, thromboelastometry.
